# Effects of Bacterial Supplementation on Black Soldier Fly Growth and Development at Benchtop and Industrial Scale

**DOI:** 10.3389/fmicb.2020.587979

**Published:** 2020-11-24

**Authors:** Emilia M. Kooienga, Courtney Baugher, Morgan Currin, Jeffery K. Tomberlin, Heather R. Jordan

**Affiliations:** ^1^Department of Biology, Mississippi State University, Starkville, MS, United States; ^2^Texas A&M AgriLife Research, Department of Entomology, Texas A&M University, College Station, TX, United States

**Keywords:** black soldier flies, microbiome, bacterial supplementation, benchtop scale, industrial scale

## Abstract

Historically, research examining the use of microbes as a means to optimize black soldier fly (BSF) growth has explored few taxa. Furthermore, previous research has been done at the benchtop scale, and extrapolating these numbers to industrial scale is questionable. The objectives of this study were to explore the impact of microbes as supplements in larval diets on growth and production of the BSF. Three experiments were conducted to measure the impact of the following on BSF life-history traits on (1) *Arthrobacter* AK19 supplementation at benchtop scale, (2) *Bifidobacterium breve* supplementation at benchtop scale, and (3) *Arthrobacter* AK19 and *Rhodococcus rhodochrous 21198* as separate supplements at an industrial scale. Maximum weight, time to maximum weight, growth rate, conversion level of diet to insect biomass, and associated microbial community structure and function were assessed for treatments in comparison to a control. Supplementation with *Arthrobacter* AK19 at benchtop scale enhanced growth rate by double at select time points and waste conversion by approximately 25–30% with no impact on the microbial community. Predicted gene expression in microbes from *Arthrobacter* AK19 treatment was enriched for functions involved in protein digestion and absorption. *Bifidobacterium breve*, on the other hand, had the inverse effect with larvae being 50% less in final weight, experiencing 20% less conversion, and experienced suppression of microbial community diversity. For those tested at the industrial scale, *Arthrobacter* AK19 and *R. rhodochrous 21198* did not impact larval growth differently as both resulted in approximately 22% or more greater growth than those in the control. Waste conversion with the bacteria was similar to that recorded for the control. Diets treated with the supplemental bacteria showed increased percent difference in predicted genes compared to control samples for functions involved in nutritional assimilation (e.g., protein digestion and absorption, energy metabolism, lipid metabolism). Through these studies, it was demonstrated that benchtop and industrial scale results can differ. Furthermore, select microbes can be used at an industrial scale for optimizing BSF larval production and waste conversion, while others cannot. Thus, targeted microbes for such practices should be evaluated prior to implementation.

## Introduction

Global demand for food produced for human consumption is predicted to increase by 100% over the span of the next 40 years (Tubiello et al., 2014). Despite efforts to keep up, agricultural production is not predicted to meet the demand (Ray et al., 2013). Increased need for cattle and other animal proteins requires increased feed production with limited available land. The space and water needs of both livestock and production of their feed account for nearly 70% of all the land used in agricultural production (FAO, 2013). Furthermore, as both human and animal populations grow, there will be an increase in waste production. Manure, food, and agricultural waste all produce greenhouse gases and noxious odors as well as serve as potential incubators for pathogenic microbes (Mawdsley et al., 1995; Sahlstrom, 2003; Tyrrel and Quinton, 2003). Therefore, safe and effective waste management solutions must be developed.

The black soldier fly (BSF), *Hermetia illucens* (L.) (Diptera: Stratiomyidae) is one of about 2000 species of insects that is used as food or feed (Mitsuhashi, 2016). The adult BSF is not a pest when properly managed (Tomberlin and Huis, 2020). Additionally, adult BSF do not need to feed to survive and reproduce (Sheppard et al., 2002). BSF larvae (BSFL) are known as voracious feeders that consume and degrade most organic materials 55 up to 70% (Sheppard, 1983; Newton et al., 1992; Diener et al., 2011). They can degrade everything from fruit and vegetables (Nguyen et al., 2015), to animal remains (Tomberlin et al., 2005; Harnden and Tomberlin, 2016) and manure (Sheppard et al., 1994; Myers et al., 2008; Miranda et al., 2019). These wastes can then be converted into insect biomass that is rich in both proteins and fats (Liu et al., 2017).

Substitution or partial replacement of traditional diets with BSFL has had positive results. Weaned pigs fed a diet consisting of 50% BSFL showed a 9% improvement in feed efficiency (Newton et al., 1977; Sheppard et al., 1994). Similarly, a study conducted on rainbow trout showed that replacing up to 40% of the fish’s diet with BSFL showed no negative effects on both the fish’s physiology and the quality of meat, but unfortunately there were lower levels of healthy polyunsaturated fatty acids (Renna et al., 2017). BSFL have also been fed to poultry, usually because they are natural colonizers of poultry manure and have been used by farmers to help with waste management and prevent manure from becoming a pollution issue (Bradley and Sheppard, 1984; Bradley et al., 1984). In many studies, BSFL were deemed a fit substitute for soybean or corn meal feed. When used to feed broiler quail, there was no difference in yield between quail whose diet had been partially replaced with BSFL and those who ate their usual diet (Cullere et al., 2016); but they did have improved amino acid levels and increased saturated and monounsaturated fatty acids that pushed the meat toward more nutritious (Cullere et al., 2016). Another poultry study conducted with broiler chickens also found that while feeding BSFL to chickens did increase the levels of undesired fatty acids, defatting the BSFL decreased this effect (Renna et al., 2017).

BSF larvae are poised for mass production for proteins and oils as we know more about this species than any other insects that hold the same potential. Numerous companies both in the United States and abroad are attempting to rear BSFL for mass production as food, as feed, and as a waste management and conversion solution. However, the system has not been optimized for maximum production of proteins and lipids or for maximum waste degradation. The first step to their optimization is performing experiments on the benchtop, in order to determine variability and efficiency in methodology. It is important, however, to recognize differences may be found when results at a small scale are compared to those obtained at the industrial scale. Reasons for this may include the sheer number of larvae in an industrial scale, as nutrient availability and access to food for each individual will differ from the small scale. In small scale studies, larvae have less competition and easier access to their food substrate, as well as less surface area. On the industrial scale, they must compete with thousands, not hundreds, of other larvae for resources. This dynamic in turn will influence waste conversion and feeding efficiency. Similarly, moisture content and the heat of the entire system will not be the same as on the benchtop because of increased number of larvae seeking out food. Studies conducted on a small scale are important for initial results, determination of important variables, and fine-tuning methodology, but must be conducted on an industrial level before these methods can be considered for application to “real world” or to a commercial setting.

BSF larvae have been shown to decrease the amount of pathogens, such as *Salmonella enterica* (Erickson et al., 2004) and *Escherichia coli*, in their substrate (Liu et al., 2008; Lalander et al., 2013, 2015) and can become contaminated with the bacteria they encounter (Erickson et al., 2004). Furthermore, studies have shown beneficial effects through bacterial supplementation. For instance, inoculating poultry manure used to raise BSFL with a bacterium, *Bacillus subtilis*, increased larval growth (Yu et al., 2011). These studies show that BSFL are able to be influenced by microbes.

By definition, probiotics are “viable microorganisms that, when ingested, have a beneficial effect” (Havenaar and Huis In’t, 1992). In human intestinal health, probiotics are able to inhibit adherence of pathogens, compete for nutrients, and stimulate immunity (Rolfe, 2000). In insects, probiotics have been found to have beneficial effects. One study showed that *Enterococcus kuehniella* isolated from larval moth feces and orally administered to red flour beetle larvae increased infection survival rates of the beetle larvae due to the probiotic’s antimicrobial activity (Grau et al., 2017). However, in another study, bees fed sugar syrup supplemented with *Lactobacillus rhamnosus* (a commercially available probiotic) were more susceptible to disease, and had a shorter lifespan (Ptaszynska et al., 2016). The latter study underscores the importance of probiotic selection in measuring health and functional outcomes.

Bacteria also provide nutrition in the form of triglycerides and lipids that are essential for insect growth and reproduction and provide energy needed during extended non-feeding periods. This is particularly true during the larval stage where energy reserves are accumulated within the fat body to be utilized during metamorphosis. There is great diversity in the concentration of lipids present in bacterial species. For instance, oleaginous microbes have a high lipid content, which composes about 20% or more of their biomass (Meng et al., 2009). Oleaginous microbes are excellent candidate organisms for the bioprocessing of chitinous waste, such as the exoskeletons of dead adult BSF, as many possess the enzymatic machinery to break down chitin and protein. Additionally, they can synthesize and accumulate triacylglycerides, similar in composition to vegetable oils, a primary material for biodiesel production (Castro et al., 2016). In a large scale, nearly zero waste system, rearing facilities could use adult flies allowed to emerge for breeding as a portion of the media used to grow the oleaginous microbes, eliminating waste output from the system and further cementing BSFL-rearing for protein as an environmentally conscious effort.

For these reasons, we selected two oleaginous microbial species to supplement into the BSFL diet mixture: *Arthrobacter AK19* and *Rhodococcus rhodochrous 21198*. We previously conducted a benchtop scale study with *R. rhodochrous* 21198, and demonstrated that *R. rhodochrous* supplementation increased larval mass (manuscript submitted elsewhere). Both *Arthrobacter* and *Rhodococcus* species have also been investigated as a means of bacterial hydrocarbon synthesis ultimately to be used in making biofuels (Srinophakun et al., 2017). Our hypothesis for studies outlined here was that the addition of fat-rich bacteria to the feeding substrate of BSFL would increase body mass, development rate, feed-to-body mass conversion, and nutrient density. We present methodology and results of both small scale and industrial scale research where BSFL were supplemented with oleaginous microbes. Additionally, we conducted a small scale study where we supplemented BSFL diet with *Bifidobacterium breve*, a well-characterized human probiotic (Shi et al., 2016). In all studies, our objectives were to measure growth, waste conversion, and gut microbiome composition in an effort to determine the utility of bacterial supplementation in BSFL rearing and industrialization, and the role gut microbes play in BSFL diet metabolism. Results of our work demonstrate the effectiveness of bacterial supplementation to BSFL food to BSFL growth and waste conversion, and the importance of scale and probiotic choice in feeding experiments.

## Materials and Methods

### Fly Colony

Black soldier fly eggs were collected from a colony at the Forensic Laboratory for Investigative Entomological Sciences (FLIES) Facility at Texas A&M University, College Station, TX, United States. Eggs were collected in three layers of 2 × 3cm corrugated cardboard blocks placed above approximately 500 g of spent grain diet saturated with water. The cardboard was replaced daily, and cardboard containing eggs was placed in a 1 L deli cup and held in an incubator at 70% relative humidity, 27°C, and 12:12 L:D until the eggs have hatched. Larvae were shipped to Mississippi State University (MSU) Department of Biological Sciences when the larvae were 11-days-old for each of the experiments conducted at MSU, Starkville, MS, United States.

### Bacterial Growth and Collection

Both *R. rhodochrous* 21198 and *Arthrobacter AK19* were grown on Luria nutrient agar and broth at pH 6.8 and 26°C for 3 days and then collected by either scraping the plates or centrifuging the broth and collecting the pellet. All of the collected bacteria were washed in a saline solution to remove residual nutrient media. *B. breve* was grown anaerobically at 37°C on plates and collected by scraping plates.

### *Arthrobacter* AK19 Supplementation-Benchtop

Larvae were divided into replicates of 300, with each replicate placed into control or treatment containers in triplicate. A perforated plastic wrap was secured on the top of the containers to prevent escape. Larvae in control containers were fed daily with 18.00 g Gainesville diet (a standard plant based diet composed of 30% alfalfa meal, 20% corn meal, and 50% wheat bran with water; Hogsette, 1992), while treatment containers were fed 16.65 g Gainesville diet supplemented with 1.35 g (approximately 1 × 10^5^ CFU, 7.5% of diet) of *Arthrobacter* AK19. Control diets received additional water in place of a supplement to make up for the moisture difference. Initial larval weights were recorded by randomly selecting 25 larvae from the containers and weighing them, as well as initial weight of the diets. Every 24 h the larvae were separated from their feeding substrate, and the larvae and waste in the container were weighed. Containers were kept in a controlled and constant environment at room temperature. After 10 days, the experiment was stopped and the larvae and waste were immediately weighed. Waste and larvae from each replicate were collected, weighed, and dried at constant temperature (55°C) for 5 days, and for 24 h, respectively, in a MyTemp Mini Digital Incubator (Benchmark Scientific) and then weighed again. Remaining larvae and waste from immediate collection were frozen in −20°C until further analysis.

### *Bifidobacterium breve* Supplementation Experiment-Benchtop

A similar treatment plan was followed for *B. breve* as was described above, except instead of 300 larvae per cup only 100 larvae were placed into each treatment container. An identical feeding plan and percent bacteria were used. Instead of placing the bacterial supplement into each day’s diet and then feeding, *B. breve* was grown on plates anaerobically until 1% (approximately 1 × 10^6^ CFU) by weight of the total diet could be replaced with the supplement. The entire volume of diet required for a 10-days-experiment was weighed out and prepared with the appropriate volume of water in advance. The diet was placed in an anaerobic chamber, maintained by anaerobic packs that were changed out daily. The inoculum amount was added into the diet and allowed to colonize and the diet required for the entire experiment was kept at growing temperature. BSFL were left on the benchtop at room temperature in their treatment containers with a perforated plastic wrap on top of each cup to prevent escape. In order to make sure temperature was not significantly different between the control and treatment groups, the diet and water for the control groups was also kept in the same incubator and was the same temperature during feedings.

### *Arthrobacter* AK19 and *R. rhodochrous* 21198 Supplementation-Industrial Scale

The industrial scale experiments were conducted using *Arthrobacter* AK19 and *R. rhodochrous* 21198. Gainesville diet was used as a diet base where either 8 g (approximately 6 × 10^5^ CFU/g) of *R. rhodochrous* 21198 or *Arthrobacter AK19* was added to 6 kg of diet per pan (four pans per treatment or control, *N* = 16 total), stirring with gloves for 30 s to homogenize the supplement. Following this, approximately 10,000 larvae were added to each pan of either non-supplemented or supplemented. Each treatment condition (*Arthrobacte*r-supplemented, *Rhodococcus*-supplemented, and control) had four replicates. The larvae were allowed to feed constantly on the initially placed food substrate. The pans were mixed daily to ensure that temperature spikes due to composting did not occur. Every 3 days, a subset of 500 larvae was removed from the pans, weighed, and frozen for later analysis. A sample of the waste was also collected and frozen for later analysis. The experiment was carried out for 10 days. On day 10, 500 larvae from each replicate were removed, weighed, and frozen. The rest of the larvae were shifted from the waste and the total mass of the larvae, as well as the total mass of waste, was weighed. One liter of waste and 500 larvae were saved and dried for 5 days and 24 h, respectively, in a MyTemp Mini Digital Incubator (Benchmark Scientific) set at 55°C to determine moisture content.

### DNA Extraction

Subsets of larvae were surface sterilized by submerging larvae in 10% bleach solution for 2 min, followed by subsequent submersion in two separate containers of molecular grade water for 2 min. Following this, larvae were cut lengthwise using a sterile scalpel, and the gut was removed. DNA from larval guts and waste were isolated using a modified protocol of that discussed in Williamson et al. (2014), quantified by a Qubit 2.0, and purified using a Qiagen DNA clean-up kit. Genomic Larval gut DNA was extracted from all replicates and the extracts were subsequently pooled. DNA was amplified with V4 primers and suggested protocols by the Earth Microbiome Project (Thompson et al., 2017) and visualized by gel electrophoresis. Verified amplifying DNA from larval guts was sent to Michigan State University Sequencing Facility, East Lansing, MI, United States, for paired-end 16S metagenome sequencing.

### DNA Sequencing

Microbial DNA samples from larval guts were sequenced using Illumina MiSeq of 2 × 250 bp paired-end reads following 16S library construction, both performed by the Michigan State University Genomics Core Facility. The V4 hypervariable region of the 16S rRNA gene was amplified using dual indexed Illumina compatible primers 515f/806r as described by Kozich et al. (2013). PCR products were normalized using Invitrogen SequalPrep DNA Normalization plates and the products recovered from the plates pooled. This pool was cleaned with AMPureXP magnetic SPRI beads and quantified using a combination of Qubit dsDNA HS, Advanced Analytical Fragment Analyzer High Sensitivity NGS DNA and Kapa Illumina Library Quantification qPCR assays. Sequencing of the pooled amplicons was on an Illumina MiSeq v2 standard flow cell using a 500 cycle v2 reagent cartridge. Custom Sequencing and index primers were added to appropriate wells of the reagent cartridge as described in Kozich et al. (2013). Base calling was done by Illumina Real Time Analysis (RTA) v1.18.54 and output of RTA was demultiplexed and converted to FastQ format with Illumina Bcl2fastq v2.19.1.

Raw fastq files barcoded Illumina 16S rRNA paired-end reads were assembled, quality-filtered, demultiplexed, and analyzed in QIIME version 1.8.0 (Caporaso et al., 2010b). Reads were discarded if they have a quality score < Q20, contained ambiguous base calls or barcode/primer errors, and/or were reads with < 75% (of total read length) consecutive high-quality base calls. Chimeric reads were removed using the default settings in QIIME (Haas et al., 2011). After quality control, the remaining sequences were binned into OTUs at a 97% sequence similarity cutoff using UCLUST (Edgar, 2010). Assembled sequence reads were classified into operational taxonomic units (OTUs) on the basis of sequence similarity. The highest-quality sequences from each OTU cluster were taxonomically assigned using the RDP classifier (Wang et al., 2007) and identified using BLAST against reference sequences from the most current Greengenes 97% reference dataset^1^ (DeSantis et al., 2006; McDonald et al., 2012; Werner et al., 2012). Representative sequences of all OTUs were aligned to the Greengenes reference alignment using PyNAST (Caporaso et al., 2010a), and low abundance OTUs (<0.0005% of reads in the total dataset) were removed (Bokulich et al., 2013). Samples were rarefied to achieve equal coverage per sample and those samples with fewer sequences were not used in subsequent analyses.

### Sequence Archiving

Sequences were archived within the NCBI Sequence Read Archive^2^ under Accession Number: PRJNA663337.

### Quantitative PCR for Detection of *Arthrobacter* and *Rhodococcus* Over Time

Primers were designed for targeting the respective 16s regions of *R. rhodochrous* and *Arthrobacter* AK-19. Primers and Taqman probe targeting *R. rhodochrous* 21198 16s included forward primer: 5′ACGACGTCAAGTCATCATGC; reverse primer: 5′ GTATCGCAGCCCTCTGTACC; probe (VIC fluorophore): VICTATGTCCAGGGCTTCACACAMGBNFQ. Primers and Taqman probe targeting Arthrobacter AK-19 16s included forward primer: 5′ GTGGGTACGGGCAGACAGA; reverse primer: 5′ CTACGCATTTCACCGCTACA; probe (FAM fluorophore): 6FAMGTGCAGTAGGGGAGACTGGAMGBNFQ. Ten-fold dilutions were created for standards using known concentrations of *R. rhodochrous* 21198 and *Arthrobacter* AK-19. Standards were analyzed in triplicate, samples were analyzed in duplicate, and qPCR reactions were multiplexed. Conditions for qPCR included 3 μL of template, 1 μL each of forward and reverse primers (2.5 μM), 2.5 μL each of probe (0.125 nM), and 12.5 μL Environmental MasterMix (ThermoFisher). Cycling conditions included an initial melting temperature of 95°C for 3 min, following by 40 cycles of 95°C for 1 min, 55°C for 30 s, and 72°C for 45 s.

### Analyses of Microbial Diversity

Bacterial diversity of larval gut microbiomes was assessed through the Chao1 estimator and the Shannon index, calculating both indexes after subsampling with QIIME and data against the Greengenes Database, to avoid sequencing bias. Mann–Whitney or Kruskal–Wallis tests were used to test statistical significance of alpha diversity.

Relative abundance was also assessed and plotted at family level using the R vegan and phyloseq packages. Family level abundance less than 2% were not shown. Statistical significance of microbial variance between groups was analyzed using the adonis function in the vegan R package given a Bray–Curtis dissimilarity matrix of the taxonomic profiles and metadata. Adonis is a permutational multivariable analysis of variance (PERMANOVA) using distance matrices. Statistical significance of the fit was assessed using 99,999 permutation tests. Bonferroni correction was used when necessary.

### Determination of Functional Capacity Using Phylogenetic Investigation of Communities by Reconstruction of Unobserved States (PICRUSt)

In order to predict genes from the metagenome, closed-reference OTUs were obtained from the filtered reads using QIIME version 1.8.0 (Caporaso et al., 2010b). The biom-formatted OTU table was then loaded to PICRUSt on the online Galaxy version in the Langille Lab (v1.1.1), alongside the Greengenes database (last updated June 2017). PICRUSt software estimates functional potential from the community metagenome using copy normalized 16S rRNA sequencing data whose gene contents are contributing to Kyoto Encyclopedia of Genes and Genomes (KEGG) identified pathways. Functionally annotated genes that were identified were compressed into 12 general gene families. Comparisons were made between differences in annotated gene abundance from control and treatment groups to determine the percent change of treatment as compared to control groups. Only those with gene abundance at or above 25% change were considered for analysis.

## Results

### *Arthrobacter* AK19 Supplementation-Benchtop

**FIGURE 1:**
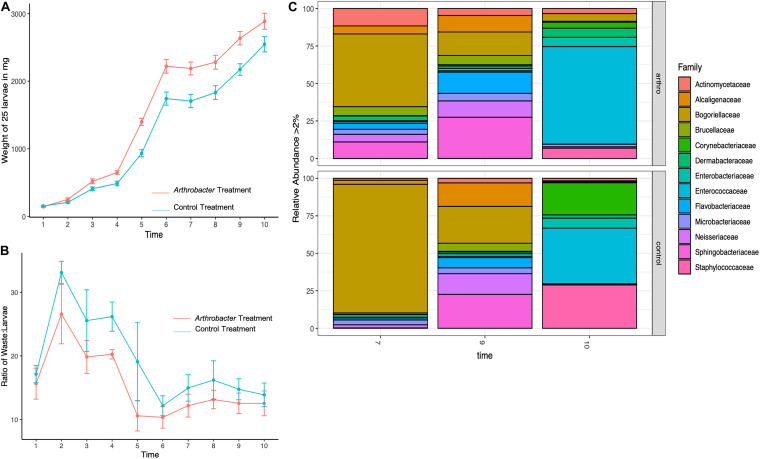
Mean daily black soldier fly larval **(A)** weights, **(B)** waste:larvae ratio, and **(C)** microbial relative abundance for *Arthrobacter* supplemented larvae compared to control at benchtop scale.

The mean daily weights for the small scale *Arthrobacter AK19* supplementation study show the mean daily weight (Figure 1A). The mean daily weight of supplemented larvae was greater than that of the control-diet larvae, particularly at early timepoints. On the third day, treatment groups were 94% larger than control in mass and had increased 107% from day 2 to day 3, whereas control larvae only increased mass by 28% from day 2 to day 3. A similar but steadily diminishing trend was seen in later timepoints: On day 5, treatment larvae were 58% larger than control larvae and increased their mass by 113% from day 4. Despite the fact that control larvae had an 85% increase in mass at the day 4 timepoint, their overall mass was still less than treatment larvae. There was a significant difference in mean daily weight between treatment and control larvae at day 3 (*p* = 0.007), day 4 (*p* = 0.0003), day 5 (*p* = 0.005), day 6 (*p* = 0.001), day 7 (*p* = 0.0006), day 8 (*p* = 0.007), day 9 (*p* = 0.002), and day 10 (*p* = 0.015).

The waste:larvae ratio was calculated for timepoints 2–10 and shows the ability of the larvae to convert their feeding substrate (Gainesville Diet) into body mass (Figure 1B). An overall lower waste:larvae ratio was observed in *Arthrobacter* supplemented groups, revealing that the bacterial supplemented larvae had an increased capacity to digest the feeding substrate and convert the substrate to biomass. The waste:larvae ratio increased during the first few days, and peaked at day 2. As the larvae continued feeding on their food and received daily feedings, the ratio, while not statistically significant (*p* = 0.793) decreased until the larvae neared the prepupal stage from T6 to T10.

**FIGURE 2:**
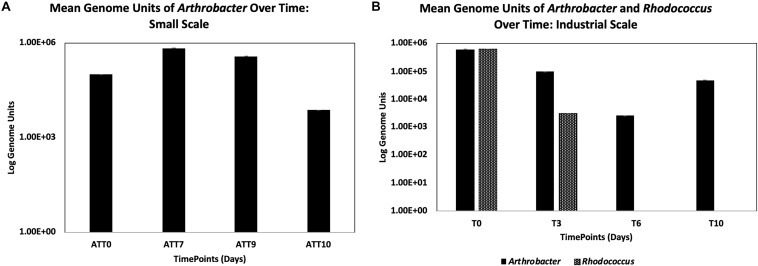
Detection of *Arthrobacter* at small scale **(A)** and *Arthrobacter* and *Rhodococcus* at industrial scale **(B)** using qPCR targeting the respective 16s genes (with standard error bars).

*Arthrobacter* was detected by qPCR in the treatment group larval guts over time (Figure 2A), where there was a slight increase from the initial inoculum within the first 7 days (from 1 × 10^5^ to 6.85 × 10^5^ CFU). *Arthrobacter* was also detected in larval guts on day 9 (3.78 × 10^5^ CFU) and on day 10, though had decreased by two logs on day 10 of the experiment (7.36 × 10^3^).

Family level microbial richness and evenness was higher in *Arthrobacter* treated larval gut microbiomes than controls on days 7 and 9 but this was reversed at day 10, at which time control samples showed an increased abundance. The difference in microbial richness and evenness between treatments at days 7, 9, and 10 were not statistically significant (*p* = 0.4).

Figure 1C shows the relative abundance of BSFL associated bacterial families. On day 7, the treatment group showed greater diversity and had increased abundance from every family represented, with the exception that the control group was composed of 39% more Bogoriellaceae and 9% more Enterococcaceae. Of those families that were increased in treatments at day 7, all showed over 100% increase except for an 11% increase in Microbacteriaceae and a 23% increase in *Staphylococcaceae*. At day 9, the differences were not as apparent. Treatment larvae saw a 21–36% decrease in Alcaligenaceae, Bogoriellaceae, and Neisseriaceae. Treatment larvae had 50% more Actinomycetaceae, 62% more Corynebacteriaceae, 44% more Dermabacteraceae, 26% more Microbacteriaceae, and over 100% increases in Flavobacteriaceae and Staphylococcaceae as compared to control larval associated microbial families. At day 10, another shift in abundance was identified. Bogoriellaceae, which was decreased from the previous timepoint, was over 300% more abundant in treatment groups. Families that saw a decrease in treatments from controls at day 10 were: Alcaligenaceae (66% decrease), Brucellaceae (21% decrease), Corynebacteriaceae (87% decrease), Enterobacteriaceae (40% decrease), Flavobacteriaceae (74% decrease), Neisseriaceae (70% decrease), and Staphylococcaceae (84% decrease). PERMANOVA of Bray–Curtis beta diversities indicated that timepoint differences explained microbial taxonomic variation (permutation test, *p* = 0.02, 99,999 permutations), where control and treatment samples were similar at days 9 and 10, but with notable differences on day 7. Treatment also explained microbial taxonomic variation as all treatments showed statistically significant microbial variation from each other (*p* < 0.001, 99,999 permutations).

PICRUSt was used to explore relationships between predicted functional gene annotations and identified metagenomes from *Arthrobacter* supplemented samples compared to controls. From this, 263 annotated genes were identified, compressed into 12 general gene families and used to compare gene abundance between treatment and control groups. Only families with gene abundance within *Arthrobacter* group at or above 25% change from control are shown in Supplementary Figure 2. Percent difference in *Arthrobacter* group gene abundance compared to control samples at 7 days revealed predicted genes enriched for functions involved in protein digestion and absorption (96.05%), Bile acid biosynthesis (82.21%), pollutant/contaminant digestion (55.82%), nucleic acid repair/replication/general metabolism (50.61%), antimicrobial metabolism/resistance (50.26%), motility and signaling (42.73%), some genes involved in lipid metabolism (42.39%), energy metabolism (44.47%), fatty acid metabolism (36.17%), amino acid metabolism (37.59%), and membrane transport (33.00%). However, compared to controls at 7 days, samples showed a decrease in functions for bile secretion (−69.69%) as well as decrease in some genes for lipid metabolism (−30.21%). At 9 days, fewer differences in gene abundance were found in the *Arthrobacter* group compared to control. Genes increased, as compared to control, included those for pollutant/contaminant degradation (43.98%), protein digestion and absorption (25.84%), and lipid metabolism (25.51%), whereas genes for functions involved in bile secretion (−54.21%) and motility and signaling (−48.15%) were decreased compared to control. At day 10, only two functional categories from the *Arthrobacter* supplemented group were increased compared to control. Those included genes involved in functions for nucleic acid repair/replication/general metabolism (−46.18%) and for bile secretion (39.05%). However, many functional categories within the *Arthrobacter* supplementation group showed a decrease in percent abundance compared to control (Supplementary Figure 2). These included genes involved in lipid metabolism (−91.59%), pollutant/contaminant degradation (−74.29%), amino acid metabolism (−59.07%), nucleic acid repair/replication/general metabolism (−52.97%), antimicrobial metabolism and resistance (−47.17%), membrane transport (−46.63%), motility and signaling (−43.15%), protein digestion and absorption (−40.59%), energy metabolism (−38.82%), and fatty acid metabolism (−31.09%).

### *Bifidobacterium breve* Supplementation Experiment-Benchtop

**FIGURE 3:**
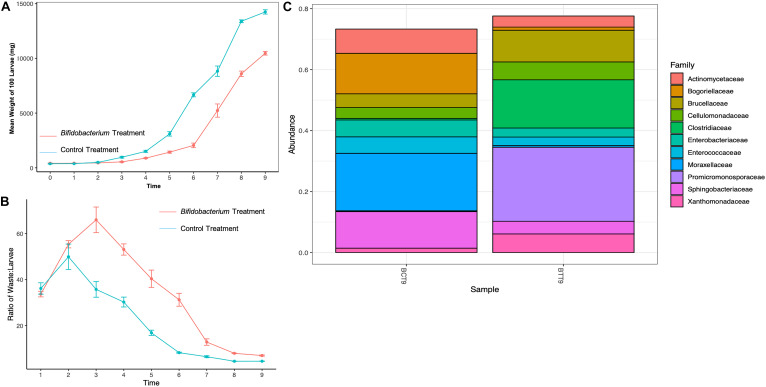
Mean daily black soldier fly larval **(A)** weights, **(B)** waste:larvae ratio, and **(C)** microbial relative abundance for *Bifidobacterium breve* supplemented larvae compared to control on day 9 of the experiment.

Supplementing with *B. breve* yielded lower weights over time compared to control BSFL (Figure 3A). Additionally, supplemented larvae appeared weak, slow, and discolored (data not shown). Also, the treatment BSFL waste:larvae ratio was much lower than control across all timepoints (Figure 3B).

Relative abundance of larval gut microbiomes from control groups showed an increased amount of Actinomycetaceae (97% increase), Bogoriellaceae (99% increase), Brucellaceae (85% increase), Cellulomonadaceae (89% increase) Enterobacteriaceae (96% increase), Enterococcaceae (96% increase), Moraxellaceae (99% increase), Sphingobacteriaceae (98% increase), and Xanthomonadaceae (72% increase) compared to treatment groups (Figure 3C). Treatment *B. breve* supplemented BSFL gut microbiomes showed an increase in Clostridiaceae (107.9% increase) and Promicromonosporaceae (510% increase) compared to controls at day 9.

PICRUSt identified 168 genes that were above 25% change from controls on day 9 of the experiment. These were compressed into 12 general gene families, as shown in Supplementary Figure 3. Samples with *B. breve* supplementation showed decrease in predicted functions for all gene families, compared to control. These included percent decrease in bile secretion (−13,601%), transport (−1,333%), protein digestion and absorption (−1,946%), pollutant/contaminant degradation (−1,958%), motility/signaling (−1,231%), lipid metabolism (−2,023%), fatty acid metabolism (−1,696%), energy metabolism (−1,491%), bile biosynthesis (−2,503%), antimicrobial metabolism/resistance (−1,491%), amino acid metabolism (−1,482%), and nucleic acid replication and repair, and general metabolism (1,488%).

### *Arthrobacter* AK19 and *R. rhodochrous 21198* Supplementation-Industrial

**FIGURE 4:**
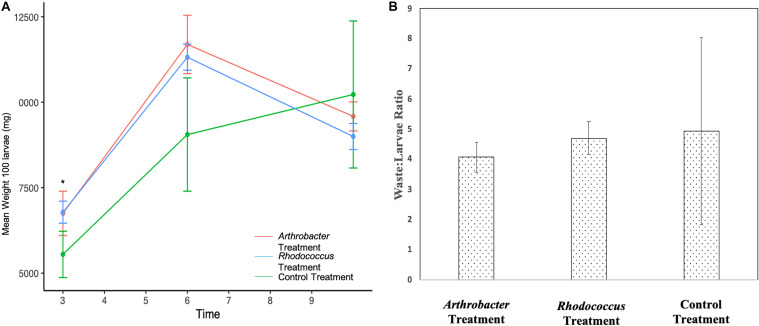
**(A)** Mean larval weight of 100 *Arthrobacter* and *Rhodococcus* supplemented black soldier fly larvae compared to control at industrial scale. Standard error bars are included. **(B)** waste:larvae ratio on day 10 across all treatments.

*Arthrobacter* and *Rhodococcus* supplemented larvae were not statistically different from each other for the duration of the study (Figure 4A). However, both treatment groups weighed statistically significantly more than control larvae at day 3 (*p* = 0.02) where *Arthrobacter* treated larvae were 21.6% larger than controls, and *Rhodococcus* treated larvae were 22.2% larger than controls. At day 6, treatment groups were not statistically different from controls, likely due to large variation in control larvae (*p* = 0.06), though *Arthrobacter* treated larvae were 29% larger than controls, and *Rhodococcus* treated larvae were 25% larger than controls. At day 10, control larvae weighed 6.3% more than *Arthrobact*er treatments, and 12.0% more than *Rhodococcus* treated larvae. The waste:larvae ratio from day 10 was not significantly different between treatments (*p* = 0.793, Figure 4B).

*Arthrobacter* and *Rhodococcus* were detected by qPCR through time to determine growth of the supplemented bacteria within the larval guts (Figure 2B). We found an initial decrease of one log (from 6.0 × 10^5^ CFU initial inoculum to 9.76 × 10^4^ CFU on day 3) from the initial *Arthrobacter* inoculum within the first 3 days of the experiment. There was continued decrease by another log by day 6 of the experiment (2.56 × 10^3^ CFU). However, 4.72 × 10^4^ *Arthrobacter* genome units were detected at day 10 (Figure 2B).

**FIGURE 5:**
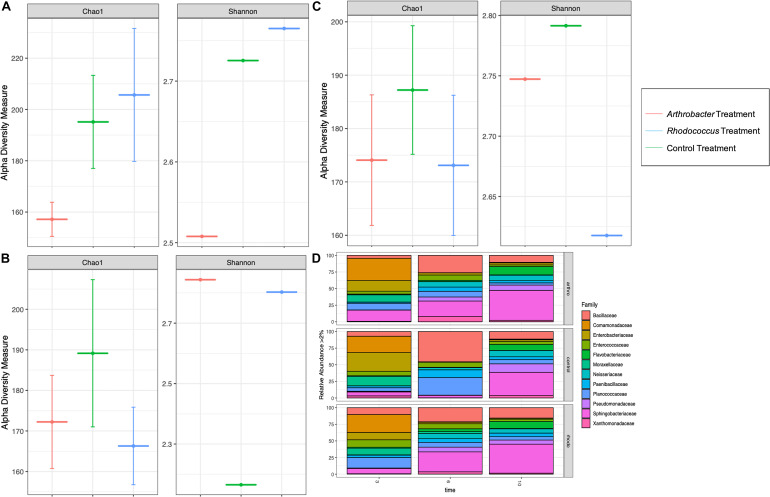
Alpha diversity measures of numbers of observed families (Chao1) and abundance and evenness (Shannon) in *Arthrobacter* and *Rhodococcus* supplemented black soldier fly larvae compared to control at industrial scale at **(A)** day 3, **(B)** day 6, and **(C)** day 10. **(D)** The microbial relative abundance of *Arthrobacter* or *Rhodococcus* supplemented black soldier fly larvae compared to control at industrial scale.

Richness was lower for larval gut microbiomes from the *Arthrobacter* treatment on day 3 than for larval gut microbiomes from the control or *Rhodococcus* treatments (Figure 5A), but was similar to *Rhodococcus* on days 6 and 10 where control richness was higher than both bacterial treatments (Figures 5B,C). The *Arthrobacter* treatment also showed lower evenness than the control and *Rhodococcus* treatments on day 3 (Figure 5A). This shifted on days 6 and 10 where both *Arthrobacter* and *Rhodococcus* treatments showed higher evenness than control (Figures 5B,C). There was no statistical significance with any of the alpha diversity metrics (*p* = 0.4).

At day 3, *Arthrobacter* and *Rhodococcus* larval gut microbiomes had similar relative abundance compared to control (Figure 5D). At day 6, compared to control, *Arthrobacter* treated larval microbiomes had 894% more Enterobacteriaceae, 12% more Enterococcaceae, 766% more Flavobacteriaceae, 157% more Neisseriaceae, 2295% more Pseudomonadaceae, 564% more Sphingobacteriaceae, 4794% more Xanthomonadaceae, 38% less Bacillaceae, 27% less Comamonadaceae, 41% less Paenibacillaceae, and 65% less Planococcaceae. Day 6 *Rhodococcus* treated larval gut microbiomes, when compared to controls had 97% more Pseudomonadaceae, 89% more Sphingobacteriaceae, 95% more Flavobacteriaceae, 24% more Enterbacteriaceae, 10% more Planococcaceae, 95% more Xanthomonadaceae, 45% Bacillaceae, 87% less Enterobacteriaceae, and 93% less Moraxellaceae. At day 10, species richness was similar in all groups. However, differences in relative abundance were noted from day 6 to day 10. For instance, the *Arthrobacter* group had notable differences at day 10 with 52% less abundance in Bacilliaceae, 72% less Planococcaceae, 72% less Entercoccaceae, 60% less Paenibacillaceae, 60% less Comamonadaceae, 34% less Enterbacteriaceae, but 35% more Moraxellaceae in controls than in *Arthrobacter* supplemented larvae (Figure 5D). Permutational analysis of variance (ANOVA) of Bray–Curtis beta diversities indicated that timepoint differences explained microbial taxonomic variation (permutation test, *p* = 0.004, 99,999 permutations), and by treatment where each sample showed statistical variance from the other (*p* < 0.001, 99,999 permutations).

**FIGURE 6:**
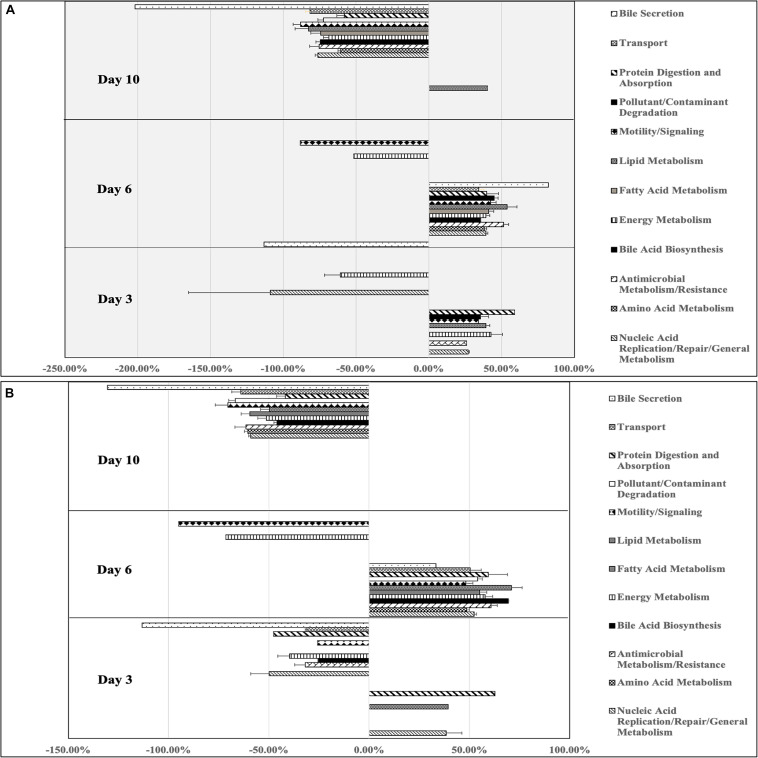
Percent differences in predicted gene functions from microbial metagenomes of black soldier fly larvae supplemented with **(A)** *Arthrobacter* or **(B)** *Rhodococcus* compared to control black soldier fly larvae at industrial scale on days 3, 6, and 10 of the experiment.

*Arthrobacter* supplemented BSF larval gut microbiomes showed increased percent difference in predicted genes compared to control samples for functions involved in protein digestion and absorption (58.93%), energy metabolism (42.77%), lipid metabolism (39.28%), pollutant/contaminant digestion (35.62%), motility and signaling (34.22%), nucleic acid replication/repair/general metabolism (27.19%), and antimicrobial metabolism/resistance (25.97%, Figure 6). Additionally, other genes for energy metabolism (−60.87%), nucleic acid replication/repair/general metabolism (−108.80%), and bile secretion (−113.19) were decreased compared to control (Figure 6A). At day 6, *Arthrobacte*r treatments showed enrichment in all general gene families, with the highest percent change from control being bile secretion (82.26%), followed by lipid metabolism (53.91%), antimicrobial metabolism and resistance (51.47%), pollutant/contaminant degradation (45.01%), motility and signaling (42.50%), fatty acid metabolism (41.29%), protein digestion and absorption (39.83%), energy metabolism (39.33%), amino acid metabolism (38.12%), bile acid synthesis (35.73%), and transport (34.42%). Only two gene families were decreased from control at day 6 including motility and signaling (−88.48%) and energy metabolism (−51.53%, Figure 6A). At day 10, *Arthrobacter* treatments only had increases in genes functionally predicted for lipid metabolism (40.41%). However, genes associated with all gene families were decreased compared to control. Those included genes for bile secretion (−201.94%), motility and signaling (−88.06%), lipid metabolism (−82.63%), transport (−81.46%), nucleic acid replication/repair/general metabolism (−76.07%), antimicrobial metabolism and resistance (−75.27%), bile acid biosynthesis (−74.67%), fatty acid metabolism (−74.36%), pollutant/contaminant degradation (−72.51%), energy metabolism (−68.67%), amino acid metabolism (−60.46%), and protein digestion and absorption (−57.99%)(Figure 6A).

At day 3, *Rhodococcus* treated BSFL microbiomes showed percent increase compared to control in genes involved in protein digestion and absorption (62.69%), nucleic acid replication/repair/general metabolism (38.38%), and lipid metabolism (39.33%, Figure 6B). Nine of the 12 gene families were decreased from control including bile secretion (−113.19%), nucleic acid replication/repair/general metabolism (−49.81%), protein digestion and absorption (−47.71%), energy metabolism (−39.69%), antimicrobial metabolism and resistance (−31.75%), transport (−31.15%), pollutant/contaminant degradation (−29.49%), motility and signaling (−25.82%), and bile acid biosynthesis (−25.60%). All gene families were increased compared to control samples, with lipid metabolism being the most increased (71.03%). Following this, bile acid biosynthesis (69.28%), antimicrobial metabolism/resistance (60.74%), protein digestion and absorption (59.38%), energy metabolism (57.88%), fatty acid metabolism (54.96%), pollutant/contaminant degradation (54.12%), nucleic acid replication/repair/general metabolism (52.21%), transport (50.32%), amino acid metabolism (48.63%), motility and signaling (48.20%), and bile secretion (33.29%). Two gene families showed percent decrease compared to control at day 6, including motility and signaling (−95%) and energy metabolism (−71.46%). On day 10, *Rhodococcus* samples showed no increased genes compared to control. Functional genes for bile secretion (−130.46%), motility and signaling (−70.40%), pollutant/contaminant degradation (−66.75%), transport (−64.09%), antimicrobial metabolism and resistance (−61.48%), amino acid metabolism (−60.46%), fatty acid metabolism (−59.57%), nucleic acid replication/repair/general metabolism (−59.06%), energy metabolism (−51.33%), lipid metabolism (−49.69%), bile acid biosynthesis (−45.87%), and protein digestion and absorption (−41.80%).

## Discussion

We hypothesized that the addition of oleaginous microbes such as *Arthrobacter* AK19 and *Rhodococcus* would increase BSFL mass. A previous benchtop scale study conducted with *R. rhodochrous* 21198 demonstrated this effect (manuscript submitted elsewhere). *Arthrobacter* AK19 was chosen for additional studies at small scale because the bacterium possesses a high concentration of lipids, usually accumulating greater than 40% lipid in dry biomass (Meng et al., 2009). With this high lipid concentration, we expected to see an amplified result in larval weight; data confirmed this expectation.

*Arthrobacter* is a well-characterized microbe commonly found in soil and in decomposition environments (Jones and Keddie, 2006). *Arthrobacter* can reduce a variety of aromatic compounds, herbicides and pesticides, hexavalent chromium and 4-chlorophenol in contaminated soil (Nordin et al., 2005), increasing interest in their use in bioremediation (see http://eawag-bbd.ethz.ch/servlets/pageservlet?ptype=allmicros for a database list of 22 *Arthrobacter* species involved in biodegrading a wide variety of compounds; Eschbach et al., 2003). *Rhodococcus* is a related genera to *Arthrobacter* and has similar biodegradation capabilities (visit http://eawag-bbd.ethz.ch/servlets/pageservlet?ptype=allmicros for a list of 30 *Rhodococcus* species involved in degrading a number of compounds). Furthermore, *Arthrobacter* and *Rhodococcus* species can degrade lignocellulosic biomass for lipid biosynthesis (Brink et al., 2019; Zhaoxian et al., 2019), and could thus be “pre-digesting” the food for the larvae, allowing an increase in nutrient availability (Jones and Keddie, 2006).

Another potential explanation is that *Arthrobacter* and *Rhodococcus* are colonizing the gut of the larvae and, like human probiotics, assisting with the digestive process. Our data indicate that *Arthrobacter* may be colonizing the gut, as we detected *Arthrobacter* by qPCR throughout the course of both studies. However, *Rhodococcus* was only detected by qPCR on day 3 of the experiment, and was below detectable limits at the remaining timepoints, suggesting only transient *Rhodococcus* passage through the gut. Another possible explanation is that *Arthrobacter* and *Rhodococcus* change the initial environmental conditions allowing other bacteria to proliferate, and may be maintained in the BSFL waste, particularly as the waste becomes more alkaline. Day 7 of the small scale study yielded percent increases in all functional groups as compared to control. This is reflective of both the taxa present as well as the relative abundance of those taxa. Unfortunately, we did not measure the microbial community within the waste in this study, nor for *B. breve*, though this is a logical next step and currently underway in subsequent studies.

Our 16s sequencing data included families where *Arthrobacter* and *Rhodococcus* reside, but detected only 184 Micrococcaceae (Family for *Arthrobacter*, data not shown) combined abundance from all treatment samples, and at all timepoints for the small scale study. We also detected Micrococcaceae from sequencing industrial scale larval guts, and found 635 combined abundance with larval gut treatments, with detected abundance at day 6 and day 10 of the study. Nocardiaceae (Family for *Rhodococcus*, data not shown) were also detected at every timepoint, with a combined abundance of 957, with decreased detection as timepoints increased. Differences in sensitivity and specificity of the two methods, along with relative abundance associated with 16s sequencing, where increase of one taxon leads to the equivalent decrease of remaining taxa, likely account for these differences (Yang et al., 2015; Jian et al., 2020).

The industrial scale experiment showed that mean daily weights of *Rhodococcus* and *Arthrobacter* supplemented larvae were consistently larger than control larvae throughout the study (Figure 3), except at the final day of the experiment. A potential explanation for the decrease in mean weights on the last day could be attributed to pupation. As the BSFL prepares for pupation, it moves into the prepupal stage. In this stage, larvae stop feeding and their integument begins to harden and darken (Lalander et al., 2019; Nyakeri et al., 2019). Their digestive system empties and they exhibit a crawl-off behavior as they seek out a safe place away from the feeding substrate where they can pupate (Dortmans et al., 2017). If the *Arthrobacter* and *Rhodococcus* supplementation was able to accelerate development, then it would potentially undergo this process sooner. In this case, when we collected samples to weigh, BSFL would have been in an advanced stage and likely weighing lighter. Additionally, there was no statistical differences in the waste:larvae ratios between the groups at industrial scale. This was not surprising since the waste:larvae ratio was only measured on day 10, where there were no differences in the larval weights for that timepoint (Figure 3A). However, because the *Arthrobacter* supplemented group still showed a better conversion ratio even at that timepoint, statistically significant differences in waste:larval ratios might have been found if measured at earlier timepoints where there was less variation between replicates.

We also note that bacterial supplementation yielded somewhat comparable results at small and large scales, depending on the timepoints. For instance, on day 3, *Arthrobacter* supplemented BSFL weighed 21% versus 22% more than control BSFL, and on day 10, 11% versus 6.7% more than control BSFL at bench and industrial scale, respectively (Figures 1A, 3A). And on day 6, *Arthrobacter* supplemented BSFL weighed 35 versus 29% more than control BSFL at benchtop versus industrial scale, respectively. Bacterial supplementation at industrial scale also yielded changes in gut microbiome species presence and relative abundance, with greatest differences found from day 6 samples, whereas large differences in gut microbiome relative abundance were also observed at day 7 or the benchtop experiment. Predicted genes involved in all functional groups were also present, similar to benchtop scale (Supplementary Figure 2 and Figure 6). But, both control and treatment larvae during both benchtop scale experiments had not reached peak weight and were still growing at the final timepoint (Figures 1A, 3A). In contrast, treatment larvae at industrial scale had reached peak weight at day 6 of the experiment with an increased number of pupated larvae (data not shown), whereas control larvae at the industrial scale were also still growing (Figure 4A). Differences in scale, numbers of larvae, amount of substrate, and differing inoculum for benchtop and industrial scale experiments likely account for this. Also, as previously mentioned, scale is important—please see the Miranda et al. (2020) reference for a discussion on this topic.

Changes in the environmental substrate during larval feeding and through supplementation likely led to changes in species composition and relative abundance. BSFL responses to stimuli including changes in water availability (Cheng et al., 2017), pH (Ma et al., 2018; Meneguz et al., 2018), temperature (Tomberlin et al., 2009), toxicity (Purschke et al., 2017), and nutrient availability (Cammack and Tomberlin, 2017) would be important in this system as these changes are observed throughout the course of larval feeding. Organisms present in high abundance in the bacterial supplemented groups appeared to have broad systems for responding to these changes including those for bacterial motility and signaling, antimicrobial resistance and biosynthesis, and pollutant/contaminant degradation. An in-depth look at predicted genes within the functional groups showed enrichment for two component systems, and higher abundance of bacterial motility and flagella proteins and bacterial secretion systems compared to control, which play important roles in bacterial attachment, colonization, and chemotaxis. Many of the identified microbial families have been found to be involved in gut digestion in mammals and other animals, as well as degradation of organic aromatic compounds and other organic pollutants (Zhang et al., 2015; Jing et al., 2020; Lavelle and Sokol, 2020). Additionally, many of these are known to produce antimicrobial and other secondary compounds.

Taken together, these results with *Arthrobacter* and *Rhodococcus* are promising and, along with previous small scale data, point toward a potential for industrialization of this process. Larvae that reach a harvestable size sooner save industrial BSF production companies money and increase their yield. If the larvae are being used for waste management, their organic material degradation ability can be increased with the aid of these probiotics, helping them to process more waste in a shorter amount of time, with even the possibility of degradation of intractable materials through bacterial supplementation directly, or indirectly through a change in microbial populations toward those with these traits.

The effect that *B. breve* supplementation had on the BSFL was unexpected. We hypothesized that addition of this bacterial species would have a positive effect on the larvae in some way, just like this probiotic can aid digestion in humans. However, this was not the case. Supplemented larvae appeared discolored, slow, covered in a sticky exudate and overall unhealthy (data not shown). They stuck to each other, to the feeding substrate, and to the container. Healthy control larvae were tan-colored, active and moved through their feeding substrate without issue. The daily mean weight of supplemented larvae was lower than the control larvae, and their waste:larvae ratio was high. *B. breve* did not aid the larvae in converting their food to body mass. Additionally, *Bifidobacterium* treatments showed an increase in Clostridiaceae, and closer inspection revealed an increase in *Clostridium* genera. Despite the increase in Promicromonosporaceae and Cellumonadaceae which contain species with high concentrations of cellulases and xylanases, digestion and frass excretion also appeared stalled. Furthermore, there was a decrease in all predicted microbial functional categories compared to controls. This result is not conducive for overall insect health if the goal is to increase growth and waste conversion. Supplementation with *B. breve* may be useful if the goal is to slow growth and development. Additionally, data from this work suggest a new mechanism for *B. breve*’s role in decreasing obesity. However, more work should be conducted to confirm this, including sampling at earlier timepoints, using differing strains and also at industrial scale.

Overall, our results show that bacterial supplementation is beneficial to BSFL larval growth and waste conversion, though care should be taken toward the appropriate bacterial supplement. We showed that bacterial supplementation yielded somewhat comparable results at small and large scales, depending on the timepoints. There was also a difference in some bacterial taxa identified among microbiomes from the two experiments. This may be due to differences in feed batches or larvae initial microbiomes, as the experiments were not conducted at the same time. It will be important to repeat the industrial scale experiments in order to determine consistency in results, particularly if bacteria are to be targeted from the results for further experimentation.

Additional studies important to the field will include the inclusion of a wide variety of food substrates including those such as spent brewer’s grain, manure, or food waste, and the inclusion of other potential probiotics. Furthermore, the effectiveness of the combination of *Arthrobacter* and *Rhodococcus* within the same treatment would be interesting. Other important data include transcriptomic data. While useful for our study, PICRUSt is limited in that genes may not be transcribed or translated, limiting the impact of their annotated function. Therefore, our conclusions about microbiome function derived from PICRUSt analyses of our metagenomes have been treated as hypotheses that require further in-depth validation through functional assays. Nevertheless, it was quite interesting that changes in functional predictions in our datasets could be related to relative abundance differences across time and treatment, based on gene annotations for a given taxa, giving insight into microbially mediated mechanisms of BSFL feeding and waste conversion. Another interesting finding was the number of taxa with functional potential for pollutant/contaminant degradation. This was an exciting finding in that there is further potential of specific bacterial supplementation, particularly many of those enriched within our studies, and manipulation in the BSFL system to allow BSFL to degrade intractable materials and also have potential utility in bioremediation, while also increasing proteins and lipids of value.

## Data Availability Statement

The datasets presented in this study can be found in online repositories. The names of the repository/repositories and accession number(s) can be found below: https://www.ncbi.nlm.nih.gov/genbank/, PRJNA663337.

## Author Contributions

EK, JT, and HJ participated in study design, conducted the study, analyzed the data, and wrote the manuscript. CB and MC aided in the setting up the study, collecting samples, and preparing samples for sequencing. All authors contributed to the article and approved the submitted version.

## Conflict of Interest

The authors declare that the research was conducted in the absence of any commercial or financial relationships that could be construed as a potential conflict of interest.

## Footnotes

1 http://greengenes.secondgenome.com

2 https://submit.ncbi.nlm.nih.gov/subs/sra/SUB8135510/overview

## Supplementary Material

Supplementary Figure 1 Alpha diversity measures of number of observed species (Chao1) and abundance and evenness (Shannon) in *Arthrobacter* supplemented black soldier fly larvae compared to control at **(A)** day 7, **(B)** day 9, and **(C)** day 10.

Supplementary Figure 2 Percent differences in predicted functions from microbial metagenomes of *Arthrobacter* supplemented BSFL compared to control black soldier fly larvae at small scale on days 7, 9, and 10 of the experiment.

Supplementary Figure 3 Percent differences in predicted functions from microbial metagenomes of *B. breve* supplemented and control black soldier fly larvae at benchtop scale on day 9 of the experiment.
