# Supplementary figures and images for: Effects of Bacterial Supplementation on Black Soldier Fly Growth and Development at Benchtop and Industrial Scale
Source: Front Microbiol. 2020 Nov 24;11:587979. doi: 10.3389/fmicb.2020.587979 (PMC7721674; doi:10.3389/fmicb.2020.587979)

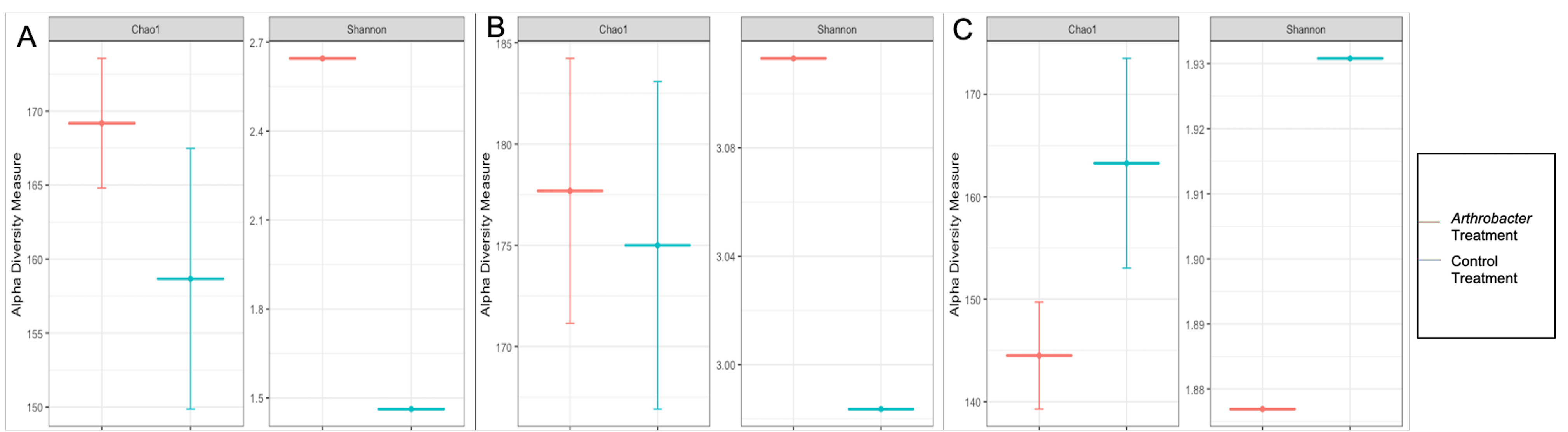

Supplement: Supplementary Figure 1 — Alpha diversity measures of number of observed species (Chao1) and abundance and evenness (Shannon) in Arthrobacter supplemented black soldier fly larvae compared to control at (A) day 7, (B) day 9, and (C) day 10. [file Image_1.tif]

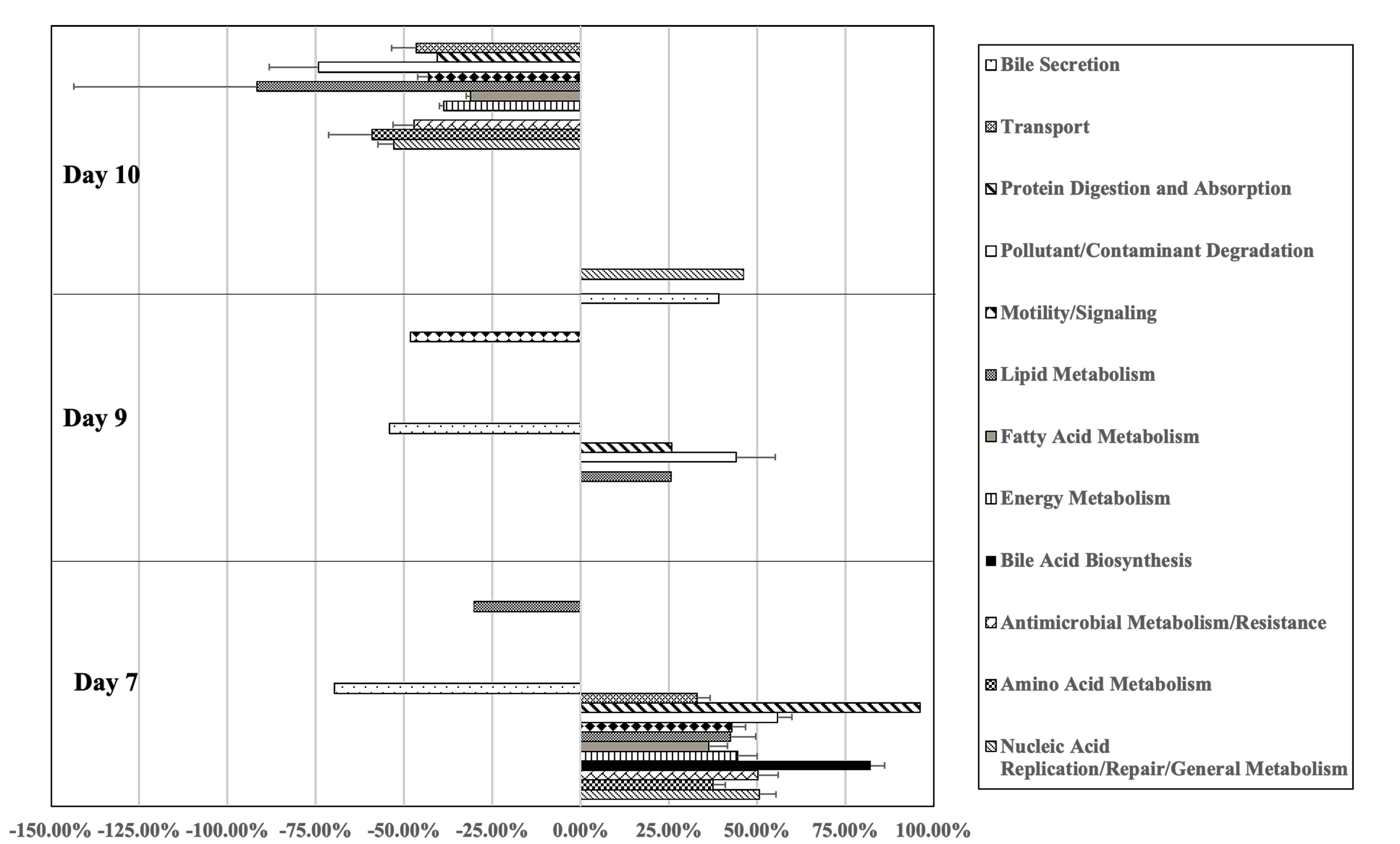

Supplement: Supplementary Figure 2 — Percent differences in predicted functions from microbial metagenomes of Arthrobacter supplemented BSFL compared to control black soldier fly larvae at small scale on days 7, 9, and 10 of the experiment. [file Image_2.tif]

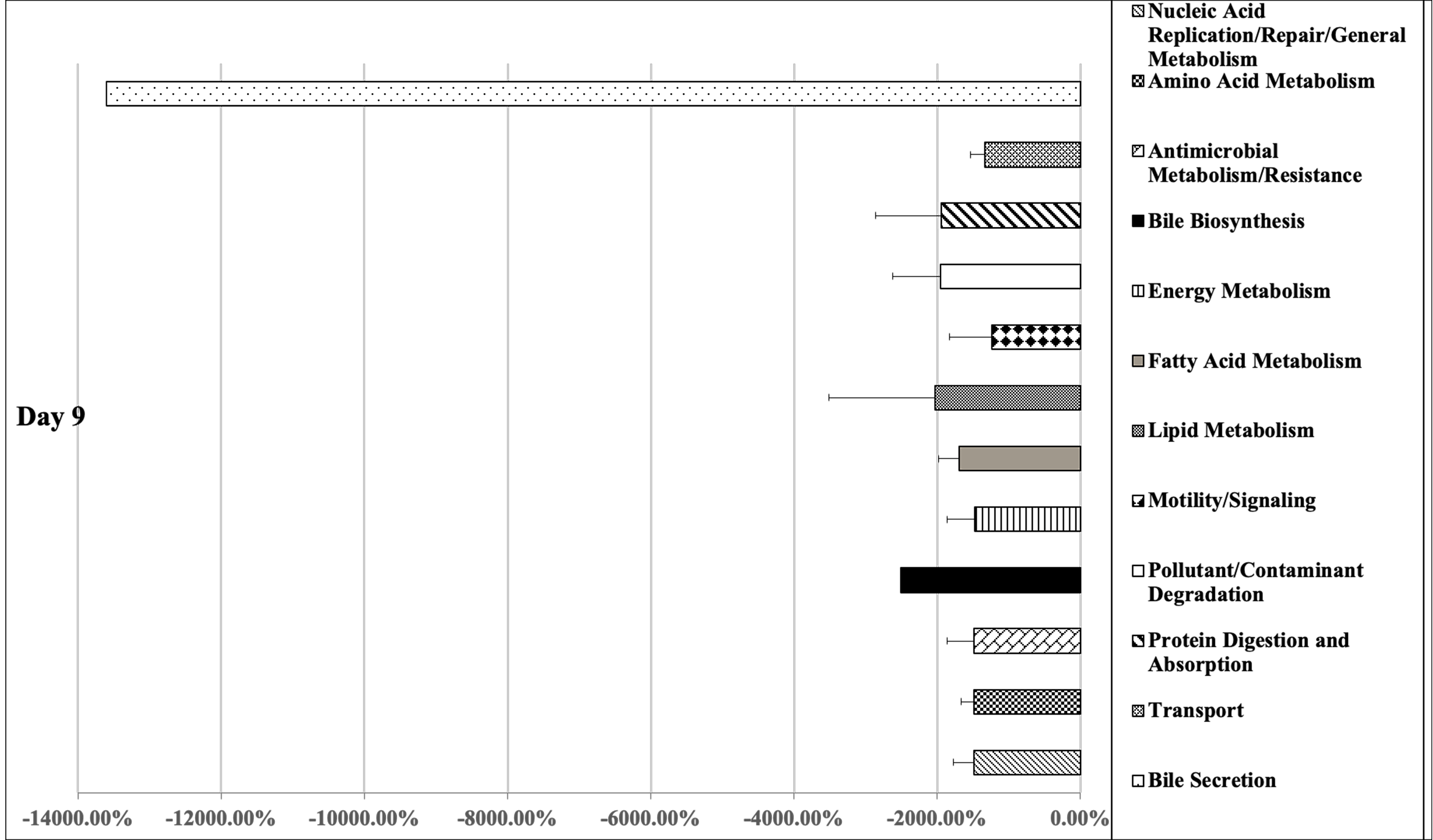

Supplement: Supplementary Figure 3 — Percent differences in predicted functions from microbial metagenomes of B. breve supplemented and control black soldier fly larvae at benchtop scale on day 9 of the experiment. [file Image_3.tif]
